# α4/α9 Integrins Coordinate Epithelial Cell Migration Through Local Suppression of MAP Kinase Signaling Pathways

**DOI:** 10.3389/fcell.2021.750771

**Published:** 2021-11-25

**Authors:** Willow Hight-Warburton, Robert Felix, Andrew Burton, Hannah Maple, Magda S. Chegkazi, Roberto A. Steiner, John A. McGrath, Maddy Parsons

**Affiliations:** ^1^ Parsons Group, Randall Centre for Cell and Molecular Biophysics, King’s College London, London, United Kingdom; ^2^ Bio-Techne (Tocris), Bristol, United Kingdom; ^3^ Steiner Group, Randall Centre for Cell and Molecular Biophysics, King’s College London, London, United Kingdom; ^4^ Department of Biomedical Sciences, University of Padova, Padova, Italy; ^5^ St Johns Institute of Dermatology, King’s College London, London, United Kingdom

**Keywords:** integrin alpha4, integrin alpha9, MAP kinase, cell movement, actin cytoskeleton

## Abstract

Adhesion of basal keratinocytes to the underlying extracellular matrix (ECM) plays a key role in the control of skin homeostasis and response to injury. Integrin receptors indirectly link the ECM to the cell cytoskeleton through large protein complexes called focal adhesions (FA). FA also function as intracellular biochemical signaling platforms to enable cells to respond to changing extracellular cues. The α4β1 and α9β1 integrins are both expressed in basal keratinocytes, share some common ECM ligands, and have been shown to promote wound healing *in vitro* and *in vivo.* However, their roles in maintaining epidermal homeostasis and relative contributions to pathological processes in the skin remain unclear. We found that α4β1 and α9β1 occupied distinct regions in monolayers of a basal keratinocyte cell line (NEB-1). During collective cell migration (CCM), α4 and α9 integrins co-localized along the leading edge. Pharmacological inhibition of α4β1 and α9β1 integrins increased keratinocyte proliferation and induced a dramatic change in cytoskeletal remodeling and FA rearrangement, detrimentally affecting CCM. Further analysis revealed that α4β1/α9β1 integrins suppress extracellular signal-regulated kinase (ERK1/2) activity to control migration through the regulation of downstream kinases including Mitogen and Stress Activated Kinase 1 (MSK1). This work demonstrates the roles of α4β1 and α9β1 in regulating migration in response to damage cues.

## Introduction

The epidermis functions as a barrier to protect against chemical, biological, and physical insults. It is primarily composed of keratinocytes (McGrath et al., 2008) which alter their physical properties as they stratify from the basement membrane towards the external environment. This process culminates in the formation of the tough and waterproof outer layer of the skin (Simpson et al., 2011). Basal keratinocytes are the only layer of the epidermis in contact with the extracellular matrix (ECM) at the dermal-epidermal junction and must therefore undergo a phenotypic switch to enable rapid collective cell migration (CCM) to reinstate the skin barrier following injury (Haeger et al., 2015).

During CCM, ‘leader’ cells at the free edge sense the external environment. This activity requires cells adjacent to the injury site to undergo partial epithelial–mesenchymal transition (EMT), to reorganize the cytoskeleton and facilitate a migratory phenotype. Leader cells also relay signals to the attached ‘follower’ cells behind the free edge. This action coordinates speed and the direction of CCM. Sustained intercellular adhesions are essential to this process, to maintain both cell polarity and contacts with neighboring cells for organized movement (Li et al., 2012). Basal keratinocytes must also undergo proliferation following injury to replenish lost cells. However, proliferation can impede migration, as the cell must detach to round and divide. Therefore, the spatio-temporal co-ordination of keratinocyte migration and proliferation is critical for effective wound closure following injury; proliferation is restricted to a region away from the migratory front, enabling keratinocytes to reinstate the population without impeding migration speed (Park et al., 2017). CCM therefore requires coordination of cell–cell and cell–ECM adhesions to maintain epidermal integrity, organize the cell cytoskeleton, and regulate signaling pathways to promote appropriate zonal control of migration and proliferation.

Integrins are the primary family of cell surface receptors to facilitate basal keratinocyte attachment to the ECM. Integrins are non-covalently attached heterodimers formed of one α and one β subunit. There are 24 known α/β combinations in mammals, created by 18 α and eight β subunits (Hynes 2002). Integrins sense ECM through the binding of short amino acid recognition sequences e.g., the RGD motif in fibronectin and laminin. ECM is bound at the cleft between integrin headpieces. Therefore, the heterodimer combination determines ligand-binding specificity. Following ECM attachment, integrins couple the ECM to the F-actin cytoskeleton to generate traction (Case and Waterman 2015). The actin cytoskeleton provides the intrinsic force to propel the cell and to control shape change during migration (Svitkina 2018). However, integrins contain no intrinsic actin binding domain or kinase activity, and therefore facilitate assembly of multi-protein intracellular adhesion complexes called focal adhesions (FA) to modulate spreading and migration (Zaidel-Bar et al., 2007; Horton et al., 2015). The proteins recruited to FA can also modulate longer-term events such as survival, growth, and proliferation through intracellular signaling pathways (reviewed in (Vachon 2011)). Therefore, integrins are important in coordinating leader and follower cell dynamics during CCM.

α4 and α9 integrins are poorly described in the context of skin biology when compared to the other α integrin mammalian subunits. However, α4 and α9 integrins have both previously been associated with epithelial tissue repair; α4 and α9 integrins bind to components of the provisional ECM (Yokosaki et al., 1999; Greena et al., 2001; Liao et al., 2002), and the expression level of both integrins increases following injury (Häkkinen et al., 2000; Singh et al., 2004; Chung et al., 2018). Furthermore, α4 and α9 integrins are known to promote a migratory phenotype during epidermal injury repair (Liao et al., 2002; Singh et al., 2004). Similarly, studies of migrating mesenchymal cells demonstrate that both α4 and α9 integrins regulate lamellipodia actin polymerization and maintain front-rear polarity, both of which are important during CCM (Hight-Warburton and Parsons 2019). These data suggest that α4 and α9 integrins may regulate processes associated with the migratory phase of skin repair following injury. However, previous work in epithelia has only defined the expression and localization of these integrins at the tissue scale (Singh et al., 2004; Chung et al., 2018). As such, α4 and α9 integrin cytoplasmic binding partners and subcellular localization in keratinocytes are currently unknown.

Here, we demonstrate that α4 and α9 integrins show distinct subcellular localization under homeostatic conditions but undergo re-localization to co-localize upon wounding. This redistribution promotes keratinocyte CCM. We further demonstrate that α4 and α9 integrins share common intracellular binding partners that are required to suppress local activation of extracellular signal-regulated kinase 1/2 (ERK1/2) and Mitogen and Stress activated kinase 1 (MSK1) in keratinocyte monolayers. This signaling leads to coordinated actin protrusion and efficient directed migration. Our data therefore provide new insight into α4 and α9 integrin functions in epithelial homeostasis and repair.

## Methods

Antibodies: Primary antibodies used were ERK1/2 (Cell Signaling Technology, Danvers, MA, United States), GAPDH (GeneTex, Irvine, CA, United States), GFP (MBL International Corporation, Woburn, MA, United States), Heat shock cognate 71 kDa protein (Sigma-Aldrich, St. Louis, MO, United States), Importin 7 (Abcam, Cambridge, United Kingdom), Integrin α2 (Thermo Fisher Scientific, Waltham, MA, United States), Integrin α4 Western blotting (NBP1-77333, Novus Biologicals, Centennial, CO, United States), Integrin α4 immunofluorescence (NBP1-77333, Novus Biologicals or Clone # 7.2R, R&D Systems, Inc.), Integrin α9 immunofluorescence (NBP2-16972, Novus Biologicals or Clone #Y9A2, Abcam), α9 integrin Western blotting (Clone # 3E4, Abnova, Taipei, Taiwan), α9 integrin function blocking (Clone #Y9A2, Bio-Rad Laboratories, Inc. Hercules, CA, United States), Integrin β1 (AB 1952, Merck Millipore, Burlington, MA, United States), Integrin β1 active (Clone # 12G10, Santa Cruz Biotechnology, Inc. CA, United States), MSK1 (R&D Systems, Inc. MN, United States), p53 (Cell Signaling Technology), Paxillin (Thermo Fisher Scientific and BD Bioscience, Franklin Lakes, NJ, United States), T202/Y204 phosphorylated ERK1/2 (Cell Signaling Technology), S380/S386/S377 phosphorylated RSK1/2/3 (R&D Systems, Inc.), S376/S360 phosphorylated MSK1/2 (R&D Systems, Inc.), RSK1 (R&D Systems, Inc.), Talin (Sigma-Aldrich), TNFR1 (Cell Signaling Technology), anti-species Horseradish Peroxidase (HRP)‒conjugated secondary antibodies (Dako, Glostrup, Denmark), AlexaFluor-conjugated antibodies (Thermo Fisher Scientific), Phalloidin (Thermo Fisher Scientific), and Hoechst (Sigma-Aldrich).

CDNA constructs: Integrin alpha4 EGFP-N3 was used, as described in (Parsons et al., 2008). Integrin alpha9 EGFP-N3 was a gift from Dean Sheppard (Addgene plasmid #13600). To generate integrin α9-TagRFP, a full-length clone of human α9 integrin in EGFP-N3 was used as a backbone (Addgene plasmid #13600). For the generation of α9-TagRFP, the coding region of the TagRFP was removed from TNFR1-TagRFP (Morton et al., 2019) using Kpn1 at the 3′ end and Not1 at the 5′ end. These sites were used to subclone TagRFP into EGFP cassette to give α9-pTagRFP-N3. Phusion cloning was carried out using Phusion® High-Fidelity PCR Master Mix with HF Buffer.

Tenascin-C and Tenascin-C (RAA) production: Glutathione S-transferase fusion proteins containing either wild type Tenascin-C or variant Tenascin-C (RAA) (a kind gift from Yasuyuki Yokosaki) were prepared by *E.coli* expression of the pGEX plasmids (Yokosaki et al., 1994); transformed bacteria were incubated until *A*_600_ reached 0.3–5 at 30 °C, at which time isopropyl-1-thio-β-galactopyranoside was added to a final concentration of 0.1 mM. Cultures were grown for several more hours, before cells were collected and sonicated (Sonics VC70T, 2 min with 10 s pulses at 15% amplitude). Glutathione S-transferase fusion proteins were affinity-purified with glutathione-Sepharose 4B beads (GE Healthcare, Chicago, IL, United States) and then cleaved off from glutathione S-transferase with PreScission Protease (GE Healthcare).

Cell culture: HPV16 immortalized human keratinocytes (NEB-1) (Morley et al., 1995) were maintained in RM + media (Fahmy et al., 1993), composed of high glucose Dulbeccos modified eagle medium (DMEM, Sigma-Aldrich) supplemented with 30% (v/v) Ham’s F12 Nutrient Mixture (Sigma-Aldrich), 10% (v/v) Fetal Bovine Serum (Thermo Fisher Scientific), 2 mM l-Glutamine (Sigma-Aldrich), 100 unit/mL penicillin and 0.1 mg/ml streptomycin (Sigma-Aldrich), 1x RM + containing 40 μg/ml hydrocortisone (Sigma-Aldrich), 500 μg/ml insulin (Sigma-Aldrich), 1 μg/ml EGF (PeproTech, Rocky Hill, NJ, United States), 0.84 μg/ml cholera toxin (Sigma-Aldrich), 500 μg/ml transferrin (Sigma-Aldrich) and 1.3 μg/ml Lyothyronine (Sigma-Aldrich). Keratinocytes were maintained at 37 °C in a 5% CO_2_ humidified atmosphere.

CDNA Transfection: Nucleoporation was used to transfect keratinocytes with cDNA. Briefly, 5 × 10^6^ cells were resuspended in 100 µl of Nucleofector® Solution (Lonza, Basel, Switzerland). Plasmids were added to cell suspension, mixed, and then transferred to a cuvette. Nucleofector® Program U-020 (Amaxa Nucleofector® II 20800900, Lonza) was used to transfect the cells.

SIRNA transfection: Keratinocytes were transfected with either a siRNA pool targeting MSK1 (GE Healthcare Dharmacon, Inc. Lafayette, CO, United States) or a non-targeting control siRNA pool (GE Healthcare Dharmacon, Inc.). Cells were incubated for 6–8 h at 37°C with the transfection solution containing siRNA and DharmaFECT (GE Healthcare Dharmacon, Inc.) in Opti-MEM (Thermo Fisher Scientific) before the media was aspirated and replaced with normal growth media. Cells were incubated for 48 h prior to experimental use.

Treatments: To inhibit α4 and α9 integrins simultaneously, keratinocytes were treated with 5 μM N-(Benzenesulfonyl)-L-prolyl-L-O-(1-pyrrolidinylcarbonyl) tyrosine sodium salt (BOP, Tocris, Bristol, United Kingdom) or 1 µM LDV peptide (Tocris) for up to 48 h 5µM methyl-BOP (MBOP, Tocris) was used as an inactive BOP control in these experiments. To inhibit α4 integrin alone, keratinocytes were treated with 10 µM BIO1211 (Tocris) for up to 48 h. To track internalized α4 integrin with time-lapse microscopy, keratinocyte monolayers transiently expressing GFP tagged α4 integrin were treated with 5 µM BOP conjugated to Janelia Fluor® 646 (BOP-JF646, Tocris). To ascertain integrin stability, keratinocytes were treated with 10 μg/ml cycloheximide (Sigma-Aldrich), 100 μM Leupeptin (Bachem Holding AG, Bubendorf, Switzerland), and 20 μM MG132 (Tocris) for up to 48 h. To inhibit ERK1/2 signaling, keratinocytes were treated with 10 μM U0126 (Tocris) for up to 1 h. To inhibit MSK1, keratinocytes were treated with 5 µM RMM46 (Tocris) for up to 24 h. To activate α9 integrin, keratinocytes were treated with 10 μg/ml Tenascin-C (RAA) for up to 4 h, and 10 μg/ml Tenascin-C was used as a control in these experiments. To inhibit importin activity, keratinocytes were treated with 25 µM Ivermectin (Sigma-Aldrich), or 50 µM Importazole (Cayman Chemical, Michigan, United States) for up to 1 h.

Fluorescence Polarization: Measurements were performed on a BMG Labtech PolarStarOmega plate reader (BMG LABTECH Ltd., Buckinghamshire, United Kingdom) at 27°C by incubating 60 nM BOP conjugated to Janelia Fluor® 549 (BOP-JF549, Tocris) with the indicated integrins (Bio-Techne) in the concentration range 0–500 nM in PBS, pH 7.4 supplemented with 1 mM MgCl_2_ and 1 mM CaCl_2_. Estimation of equilibrium dissociation constant (*K*_D_) values was performed assuming a one-site specific-binding model using the Prism package (GraphPad Software, San Diego, CA, United States). Data points are the mean of three replicates.

GFP-TRAP® Immunoprecipitation: Keratinocytes transiently expressing GFP-tag proteins were lysed in cold GFP TRAP lysis buffer (50 mM Tris (pH 7.4) (Sigma-Aldrich), 150 mM NaCl (Sigma-Aldrich), 1% NP40 (Sigma-Aldrich), 15 mM MgCl_2_ (Sigma-Aldrich), 10% glycerol (Sigma-Aldrich), 1% Triton X-100 (Thermo Fisher Scientific), 5 mM EDTA (Sigma-Aldrich), Protease inhibitor cocktail set 1 (Merck Millipore)) was added to the cells. After a 10-min incubation on ice, keratinocytes were scraped and centrifuged for 10 min at 4°C. Supernatant was then incubated with 1:1 of GFP-TRAP® A beads (Chromotek, Planegg-Martinsried, Munich, Germany) and control agarose resin (Thermo Fisher Scientific) for 2 hours. Beads were then washed and 2x sample buffer (60 mM Tris-HCl (pH 6.8) (Sigma-Aldrich), 25% Glycerol (Sigma-Aldrich), 2.5% SDS (Sigma-Aldrich), 0.02% Bromophenol blue (Sigma-Aldrich), 2% β-mercaptoethanol (Sigma-Aldrich)) was added to the beads. Samples were boiled for 5 minutes at 95°C, and then subjected to either silver stain or Western blot analysis.

Liquid chromatography with tandem mass spectrometry analysis (LC-MS/MS): GFP-TRAP complexes were fully resolved on a SDS PAGE gel. Bands of interest were excised and incubated with 10 mM dithiothreitol at 56°C and then alkylated with 55 mM iodoacetamide at room temperature. Samples were digested using 1:20 (enzyme:substrate) ratio of bovine trypsin incubated in a shaking heat block at 37°C for 16 h. Peptides were extracted with aqueous dehydration/hydration using acetonitrile and 50 mM triethylammonium bicarbonate, pooled and dried. Samples were resuspended in 2% (v/v) acetonitrile, 0.05% (v/v) formic acid and peptides were resolved by reversed phase chromatography on a 75 μm C18 Pepmap column (50 cm length) using a three-step linear gradient of 80% acetonitrile in 0.1% formic acid (U3000 UHPLC NanoLC system; ThermoFisherScientific, United Kingdom). The gradient was delivered to elute the peptides at a flow rate of 250 nl/min over 60 min starting at 5% B (0–5 min) and increasing solvent to 40% B (5–40 min) prior to a wash step at 99% B (40–45 min) followed by an equilibration step at 5% B (45–60 min). The eluate was ionized by electrospray ionization using an Orbitrap Fusion Lumos (ThermoFisherScientific, United Kingdom) operating under Xcalibur v4.1.5. The instrument was programmed to acquire in automated data-dependent switching mode, selecting precursor ions based on their intensity for sequencing by collision-induced fragmentation using an Orbitrap-Ion Trap method defining a 3s cycle time between a full MS scan and MS/MS fragmentation. Orbitrap spectra (FTMS1) were collected at a resolution of 120,000 over a scan range of m/z 375–1,500 with an automatic gain control (AGC) setting of 4.0e5 with a maximum injection time of 35 ms. Monoisotopic precursor ions were filtered using charge state (+2–+7) with an intensity threshold set between 5.0e3–1.0e20 and a dynamic exclusion window of 35s ±10 ppm. MS2 precursor ions were isolated in the quadrupole set to a mass width filter of 1.6 m/z. Ion trap fragmentation spectra (ITMS2) were collected with an AGC target setting of 1.0e4 with a maximum injection time of 35 ms with CID collision energy set at 35%. Raw mass spectrometry data were processed using Proteome Discoverer (v2.2; ThermoFisherScientific) to search against the Uniprot Human Taxonomy database (49,806 entries) with Mascot (v2.6.0; www.matrixscience.com) and Sequest search algorithms. Precursor mass tolerance was set to 20 ppm with fragment mass tolerance set to 0.8 Da with a maximum of two missed cleavages. Modifications: Carbamidomethylation (Cys) was set as fixed and Oxidation (Met) set as variable. Processed LC-MS/MS data files produced database generated files (msf) which were uploaded into Scaffold 4 (v4.10.0) software (www.proteomesoftware.com) to create a.sfd file. The raw data was searched at a stringency threshold of 1% false discovery rate (FDR) for protein and a minimum of one peptide per protein as determined by Mascot and Sequest in the Proteome Discoverer method. The peptide threshold was set to 95%.

The mass spectrometry proteomics data have been deposited to the ProteomeXchange Consortium via the PRIDE (Perez-Riverol et al., 2019) partner repository with the dataset identifier PXD027944 and 10.6019/PXD027944.

Phospho-kinase antibody array: Keratinocytes were treated with integrin inhibitors BOP, BIO1211, LDV, and DMSO as a control for either 0.5 or 6 h before being subjected to Human Phospho-Kinase Antibody Array (R&D Systems, Inc.) as per manufacturer’s instructions.

Western blotting: Western blotting was used for specific protein detection following SDS-PAGE. The proteins were transferred onto PVDF membranes (Sigma-Aldrich) then blocked using 5% BSA/TBST (0.1% Tween (Sigma-Aldrich) in Tris Buffered Saline (TBS)) or 5% milk/TBST for 1 h at room temperature. The membranes were then probed with the primary antibody at 1:1,000 dilution overnight at 4°C. The membranes were then washed prior to incubation with HRP-conjugated secondary antibodies for 1 h at room temperature. Proteins were then detected with ECL chemiluminescence kit (Bio-Rad Laboratories) and imaged (ChemiDoc Imaging Systems, Bio-Rad Laboratories). Blots were analyzed and processed using Image Lab (v5.2.1, Bio-Rad Laboratories).

Immunofluorescence and confocal microscopy: Coverslips were coated with Human Plasma Fibronectin Purified Protein (Merck Millipore). Keratinocytes were plated on coverslips either in RM + media, or RM + media without EGF supplement to force colony formation. Once at the correct confluency, cells were treated over variable time points. Cells were then washed and fixed with 4% paraformaldehyde (Sigma-Aldrich) in PBS before 10-min permeabilization with 0.2% Triton X-100, followed by blocking with 5% Bovine Serum Albumin (BSA, Roche, Basel, Switzerland) in PBS for 30 min. The cells were labeled with antibodies at 4°C overnight before being stained with secondary antibodies, phalloidin-AlexaFluor, and Hoechst for 1 h at room temperature. The cells were then mounted using Fluorsave Mounting Media (Calbiochem, San Diego, CA, United States) and imaged with a 60x objective using Nikon A1R inverted confocal microscope (Nikon, Minato City, Tokyo, Japan). Images were exported from the Nikon Elements software (Nikon) for further analysis in ImageJ software (United States National Institutes of Health, Bethesda, MD, United States).

Single cell migration assay: Cells were plated in 12 well tissue culture plates containing 1 ml RM + media (1 × 10^4^ cells per well) and allowed to adhere for 6 h at 37°C. Media was then replaced with RM + containing 25 µM HEPES and treatment. Cells were imaged live using IX71 inverted microscope (Olympus, Shinjuku City, Tokyo, Japan) every 15 min for 24 h. TIFF files were imported into ImageJ software (United States National Institutes of Health), where cells were tracked using TrackMate plugin. Data were then imported into Chemotaxis Tool (Ibidi, Gräfelfing, Munich, Germany) for analysis.

Collective cell migration assay: Keratinocytes were plated at confluency and incubated for 2–24 h in the presence of 2 mM of calcium. Confluent monolayers were wounded with a 10 µl pipette tip, and then washed to remove cell debris. Media was then replaced with RM + containing 25 µM HEPES and treatment. Cells were imaged live using IX71 inverted microscope (Olympus) every 15 min for 24 h and were saved as TIFF files. Space between two migration fronts was used as a measure of collective migration. Wound closure was analyzed using ImageJ software (United States National Institutes of Health) a percentage closure at a set time point compared to 0 h. Experiments were performed in the presence of Mitomycin C to control for proliferation changes contributing to migration. No differences were seen between control and Mitomycin C treated cultures in terms of migratory response (not shown).

Proliferation assay: Keratinocytes were plated in RM + media 1 day before the experiment. The cells were then treated with conditioned media containing inhibitors for 24 and 48 h. Cells were then fixed and stained with Hoechst and Phalloidin, before being imaged by tile-scan using EVOSTM FL Auto 2 Imaging System (Thermo Fisher Scientific) with a 10x objective lens. The number of cells in each well was measured by automated nuclei counting using ImageJ software (United States National Institutes of Health).

Colony protrusion assay: Keratinocyte colonies were treated with integrin inhibitors for 24 h to induce protrusion formation. Colonies were then co-treated for 1 h, fixed and stained with Hoechst and Phalloidin. Cells were imaged using EVOS™ FL Auto 2 Imaging System with a 20x objective lens. The percentage increase in area and perimeter of the protrusion from keratinocyte colonies was calculated by subtracting the region enclosed by the cortical actin ring from the binarized total boundaries of the cluster area using ImageJ software (United States National Institutes of Health).

Image analysis: All images were analyzed using ImageJ software and associated plugins (United States National Institutes of Health). To analyze protein signal at the cell periphery or at the leading edge, regions of interest (ROI) were generated to extract the first 5 µm behind the cell boundary. Manders overlap coefficient (MOC) of ROI was calculated using the JACoP plugin. ROIs generated using this method were also used to calculate percentage of internalized α4 integrin in follower cells (cells behind the first two rows adjacent to a scratch); the percentage of internalized α4 integrin = (signal in 5 µm reduced ROI/total signal in ROI around cell periphery) x 100. α9 integrin signal in leader cells was calculated by measuring the basal α9 integrin signal in the first 60 µm behind the leading edge in scratch assays. Nuclear MSK1 signal leader (first 2 rows of cells adjacent to scratch) was calculated by measuring the mean grey value of ROIs generated using the nuclei-stained channel. To count the number of protrusions that extended beyond the leading edge during CMM following inhibitor treatment, protrusion that extended beyond the leading edge during the first 6 h of image acquisition were measured; protrusions that extended more than 20 µm from the leading edge were counted.

Statistical analysis: Data are represented as the mean ± standard error of the mean (s.e.m.). All statistical tests were carried out using Prism package (GraphPad software). The Student’s t-test was performed for comparing two groups for statistical analysis. Analysis of variance (ANOVA) with Sidak post hoc test was used for multiple comparisons. *p* < 0.05 was considered as statistically significant.

## Results

### α4β1 and α9β1 Integrins Localize to the Leading-Edge During Keratinocyte Collective Cell Migration

**FIGURE 1:**
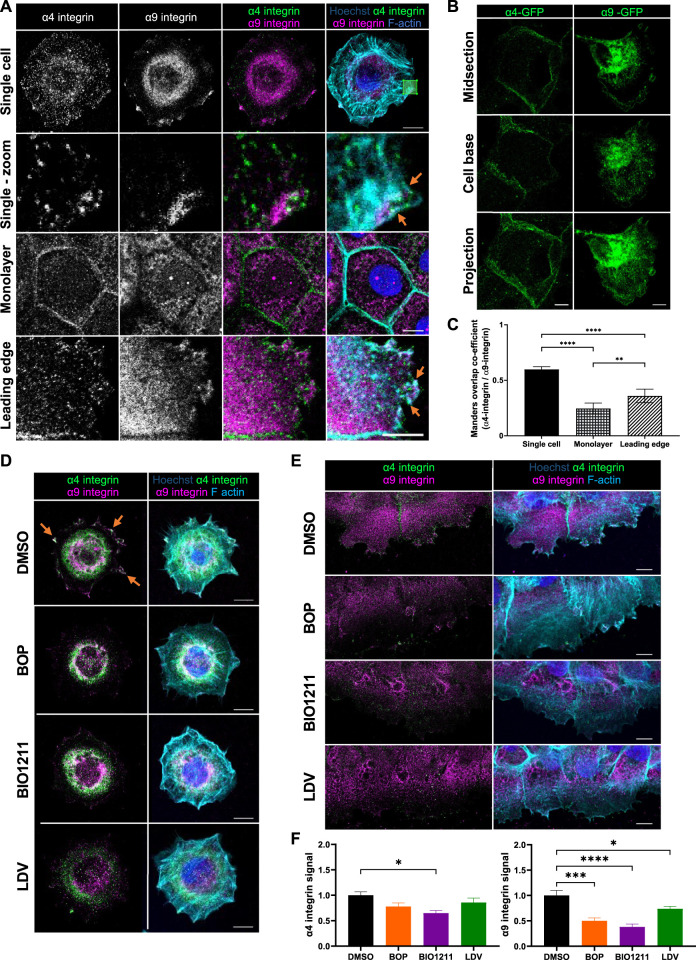
α4β1 and α9β1 localize to different subcellular compartments in keratinocytes. **(A)** Single cell: single confocal Z image at the basal plane of single keratinocytes plated on fibronectin for 0.5 h. White box indicates magnified region in panels below. Monolayer: single confocal Z image in the central plane of confluent Ca^2+^ treated keratinocyte monolayer. Leading edge: single confocal Z image at the basal plane of the leading edge of monolayer scratched with pipette tip 1.5 h prior to fixing. Cells were then stained for nuclei, α4 integrin, α9 integrin, and F-actin. Scale bar = 10 μm. Images representative of three independent experiments. Orange arrows indicate colocalization at free/leading edge. **(B)** Confluent monolayers of keratinocytes transiently expressing GFP tagged α4 integrin (α4-GFP) or GFP tagged α9 integrin (α9-GFP) for 48 h were scratched with pipette tip 1.5 h prior to fixing. α4-GFP shows cells in intact monolayer. α9-GFP shows cell at leading edge. Midsection: single confocal Z image in the central plane of confluent Ca^2+^ treated keratinocyte monolayer. Cell base: single confocal Z image at base of confluent Ca^2+^ treated keratinocyte monolayer. Projection: Maximum intensity projection of 20 μm Z stack of keratinocytes. Scale bar = 10 μm. **(C)** MOC was calculated for α4 integrin and α9 integrin. Data pooled from 30 cells per condition from three independent experiments showing mean ± s.e.m; **p < 0.01, ****p < 0.0001. **(D)** Single confocal Z image at the basal plane of keratinocytes plated on fibronectin for 0.5 h before being treated with vehicle control (DMSO), BOP, BIO1211, or LDV for 1 h prior to fixing. Cells were then stained for nuclei, α4 integrin, α9 integrin, and F-actin. Scale bar = 10 μm. Images representative of one independent experiment. **(E)** Single confocal Z image at the basal plane of keratinocytes in scratch assay. Confluent Ca^2+^ treated keratinocyte monolayers were scratched with a pipette tip 1 h prior to treatment with vehicle control (DMSO), BOP, BIO1211, or LDV for 1 h. Fixed cells were stained for nuclei, α4 integrin, α9 integrin, and F-actin. Scale bar = 10 μm. Images representative of three independent experiments. **(F)** Quantitative analysis of α4 and α9 integrin signal at the leading edge. Data pooled from 15 fields of view per condition from three independent experiments showing mean ± s.e.m.; *p < 0.05, ***p < 0.001, ****p < 0.0001. All other comparisons were not significant.

To understand whether α4 and α9 integrins are spatially co-located, we firstly analyzed the localization of each subunit in human skin and in immortalized keratinocytes. α4 integrin also heterodimerizes with β7 integrin, but β7 is not expressed in keratinocytes (Rippa et al., 2013; Watt and Jones 1993) and so α4 integrin staining in these cells only shows α4β1 integrin. α4 and α9 integrin localized to the dermal-epidermal junction in human skin (Supplementary Figure S1A,B). Moreover, alterations to α9 integrin localization in human epidermis (Supplementary Figure S1B) suggested keratinocyte adhesion state may influence α9 integrin subcellular distribution. However, the locations of α4β1 and α9β1 in keratinocytes during adhesion and migration were undefined. To address this, cells were either plated as single cells for 0.5 h on plasma fibronectin (to mimic the provisional matrix during wound healing, and analyze localization during early cell spreading), as calcium treated monolayers (to investigate localization in intact epithelial sheets following junction formation), or after inducing a scratch wound in calcium treated monolayers (to study localization at the leading edge of collectively migrating cells). Images obtained from both antibody staining and expression of GFP tagged integrin complexes revealed that α4 and α9 integrins localized to distinct subcellular compartments in keratinocyte cell monolayers (Figure 1A). α4 integrin was present at intercellular junctions, whereas α9 integrin localized to reticular-like cytoplasmic structures in cultured keratinocytes (Figures 1A,B). However, both α4 and α9 integrins were enriched at the leading edge of wounded monolayers undergoing CCM; α4 integrin localized to puncta along the lamellipodia and stress fibers (Figure 1A), and colocalized with paxillin (Supp Figure 1C, top panels) comparable to previous studies using monocytes and α4 integrin over-expression in CHO cells (Pinco, et al 2002; Goldfinger et al., 2003; Rosado et al., 2011). Conversely, α9 integrin was recruited to patches along the plasma membrane (Figures 1A,B). α9 integrin appeared to colocalize with FA markers (paxillin, talin, and vinculin) at protrusive tips but not behind the leading edge (Supplementary Figure S1C,D). Recruitment of α4 and α9 integrins to the free edge of spreading single cells and to the leading edge of collectively migrating cells following wounding (orange arrows, Figure 1A) resulted in a significant increase in their colocalization (Figure 1C).

Attempts to modulate α4 and α9 integrin expression in our keratinocyte cell line using RNAi and CRISPR were unsuccessful. Instead we utilized a panel of well-characterized inhibitors that target both α4 and α9 integrin (BOP (Pepinsky et al., 2002) and LDV-FITC (LDV, a peptide mimetic of the amino acid motifs bound by α4 and α9 integrins (Chigaev et al., 2001; Njus et al., 2009)), or α4 integrin specifically (BIO1211 (Lin et al., 1999; Muro et al., 2009)) to determine whether this re-localization was dependent upon α4 and α9 integrin activity. A fluorescence polarization assay (FP) was used to validate the relative binding affinities of α4 and α9 integrin to fluorophore labelled BOP (BOP-JF549). The calculated *K*_D_ values (Supplementary Figure S1E) are comparable to those previously reported (Cao et al., 2014). These data confirm that BOP is a high affinity dual α4/α9 integrin antagonist. Analysis of images revealed reduced localization of both integrins at leading edge of single cells undergoing early spreading (Figure 1D) and in wounded monolayers following inhibition of their activity (Figures 1E,F; Supplementary Figure S1C). Similarly, there was a reduction in the colocalization of both integrins with paxillin at the leading edges following treatment with BOP (Supplementary Figure S1C). Further analysis demonstrated that whilst α4/α9 inhibition induced internalization of both integrins (Supplementary Figure S2A–D), α4 and α9 integrins were not degraded during the 24-h timeframe of the experimental set-up (Supplementary Figure S2E). To investigate the stability of internalized α4 and α9 integrins, inhibitors were used to block protein synthesis and degradation. p53 was used as a positive control for proteasomal degradation (Chao 2014). TNF-R1 was used as a positive control for lysosomal degradation (Mosselmans et al., 1988). Expression of p53 and TNF-R1 was depleted within 4 h of cycloheximide treatment, indicating that protein synthesis was being blocked (Supplementary Figure S2F). p53 expression was increased after 24 h of treatment with MG132, and TNF-R1 expression was increased after 24 h of leupeptin treatment validating the inhibitors for this experiment (Supplementary Figure S2F). α4 and α9 integrin protein levels were unchanged following treatment with cycloheximide for up to 24 h (Supplementary Figure S2F). α4 and α9 integrin protein levels remained unchanged during 24 and 48 h of treatment with MG132 or leupeptin (Supplementary Figure S2F,G). Integrins have low degradation rates and therefore to compare α4 and α9 integrins to another β1 integrin containing heterodimer expressed in keratinocytes, α2 integrin protein expression was also monitored. Unlike α4 and α9 integrin, α2 integrin expression was reduced after 4 h of cycloheximide treatment compared to DMSO control cells, and this reduction in protein expression level was maintained after 24 h of treatment (Supplementary Figure S2F). The especially slow degradation of α4 and α9 integrins suggests that they are either stable at the cell surface or are readily shuttled between the cell surface and intracellular compartments to re-localize upon induction of CCM. This observation suggested that α4 and α9 integrins may co-operate to regulate processes at the leading edge that are imperative for CCM, such as actin polymerization and cell adhesion to the ECM.

### Inhibition of α4/α9 Integrins Enhances F-Actin Protrusion and Reduces Keratinocyte CCM

**FIGURE 2:**
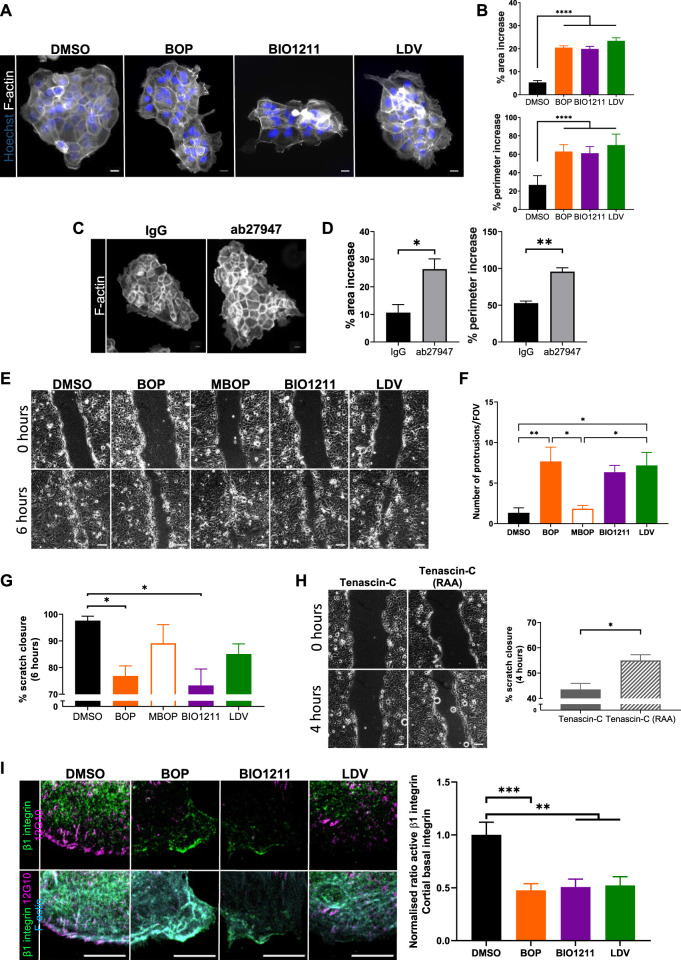
α4/α9 integrins are required for efficient keratinocyte migration. **(A)** Widefield images of Ca^2+^ treated keratinocyte colonies treated with vehicle control (DMSO), BOP, BIO1211, or LDV for 24 h prior to fixing. Cells were then stained for nuclei and F-actin. Scale bar = 20 μm. Images representative of three independent experiments. **(B)** Quantitative analysis of percentage increase in area and perimeter of actin protrusions over cortical actin ring. Data pooled from 30 colonies per condition from three independent experiments showing mean ± s.e.m.; ****p < 0.0001. **(C)** Widefield images of Ca^2+^ treated keratinocyte colonies treated with control antibody (IgG) or ab27947 (α9 integrin function blocking antibody) for 24 h prior to fixing. Cells were then stained for F-actin. Scale bar = 20 μm. Images representative of three independent experiments. **(D)** Quantitative analysis of percentage increase in area and perimeter of actin protrusions over cortical actin ring. Data pooled from 30 colonies per condition from three independent experiments showing mean ± s.e.m.; *p < 0.05, **p < 0.01. **(E)** Phase contrast images of keratinocyte scratch closure at 0 and 6 h. Confluent Ca^2+^ treated keratinocyte monolayers were scratched with a pipette tip 1 h prior to treatment with vehicle control (DMSO), BOP, MBOP (inactive BOP control), BIO1211, or LDV overnight. Scale bar = 100 μm. Images representative of three independent experiments. **(F)** Quantitative analysis of the number of actin protrusions that extended >20 µm beyond the leading edge during the 6 h time course. Data pooled from six fields of view (FOV) per condition from three independent experiments showing mean ± s.e.m.; *p < 0.05, **p < 0.01. All other comparisons were not significant. **(G)** Quantitative analysis of scratch closure after treatment. Data pooled from nine movies per condition from three independent experiments showing mean ± s.e.m.; *p < 0.05. All other comparisons were not significant. **(H)** Phase contrast images of keratinocyte scratch closure at 0 and 4 h. Confluent Ca^2+^ treated keratinocyte monolayers were scratched with a pipette tip 1 h prior to treatment with Tenascin-C (control protein), or Tenascin-C (RAA) (α9 integrin specific ligand) overnight. Scale bar = 100 μm. Images representative of three independent experiments. Quantitative analysis of scratch closure after treatment. Data pooled from nine movies per condition from three independent experiments showing mean ± s.e.m.; *p < 0.05. **(I)** Single confocal Z image at the basal plane of keratinocytes at the edge of colony. Ca^2+^ treated keratinocyte colonies treated with vehicle control (DMSO), BOP, BIO1211, or LDV for 24 h. Fixed cells were stained for nuclei, β1 integrin, active β1 integrin (12G10), and F-actin. Scale bar = 10 µm. Images representative of three independent experiments. Quantitative analysis of the ratio of active to total β1 integrin in protrusive area only. Data pooled from 15 fields of view per condition from three independent experiments showing mean ± s.e.m; **p < 0.01, ***p < 0.001.

Previous studies have shown that α4 integrins organize local F-actin assembly in migrating single fibroblasts (Ring et al., 2011). To assess whether this also occurred in keratinocytes, cell colonies were treated with α4/α9 integrin inhibitors for 24 h and then stained for F-actin. Images and quantification revealed significantly increased F-actin protrusion in cells at the edges of colonies treated with all 3 inhibitors (Figures 2A,B). Unfortunately, there is currently no α9 specific inhibitor, and so we used an α9 integrin function blocking antibody (ab27947) to assess if this phenotype could be attributed to α4 integrin function alone. Colonies treated with the α9 integrin blocking antibody also showed F-actin protrusions (Figures 2C,D) further supporting that activity of both α4 and α9 integrin subunits can suppress of F-actin based protrusions.

Suppression of aberrant actin polymerization restricts multiple lamellipodia formation (Pankov et al., 2005) and facilitates cell directionality (Dang et al., 2013; Gorelik and Gautreau 2015), both of which are required for directional persistence during migration. Scratch assays were treated with the 3 integrin inhibitors, as well as methyl-BOP (MBOP, an inactive BOP control (Venkatraman et al., 2005; Hutt, et al 2011)) to investigate if α4/α9 integrin-regulated actin polymerization contributed to directional persistence during keratinocyte CCM (Figure 2E). Quantification revealed leader cells treated with all 3 inhibitors had significantly increased F-actin protrusion compared to controls (Figure 2G), and this negatively impacted the rate of collective migration (Figures 2E,G). Conversely, exogenous addition of soluble Tenascin-C was used to activate β1 integrin containing heterodimers (Nishio et al., 2005). Specifically activating α9 integrin with addition of Tenascin-C (RAA) ligand (Yokosaki et al., 1994) increased the rate of CCM (Figure 2G). Furthermore, images and quantification active β1 integrin in colonies showed that α4/α9 integrin inhibition reduced β1 integrin activity at the periphery of keratinocyte colonies (Figure 2I). These findings suggest that α4 and α9 integrins localization and activity may be required for directional persistence by negatively regulating local F-actin polymerization.

### α4/α9 Integrins Negatively Regulate ERK1/2 Signaling

**FIGURE 3:**
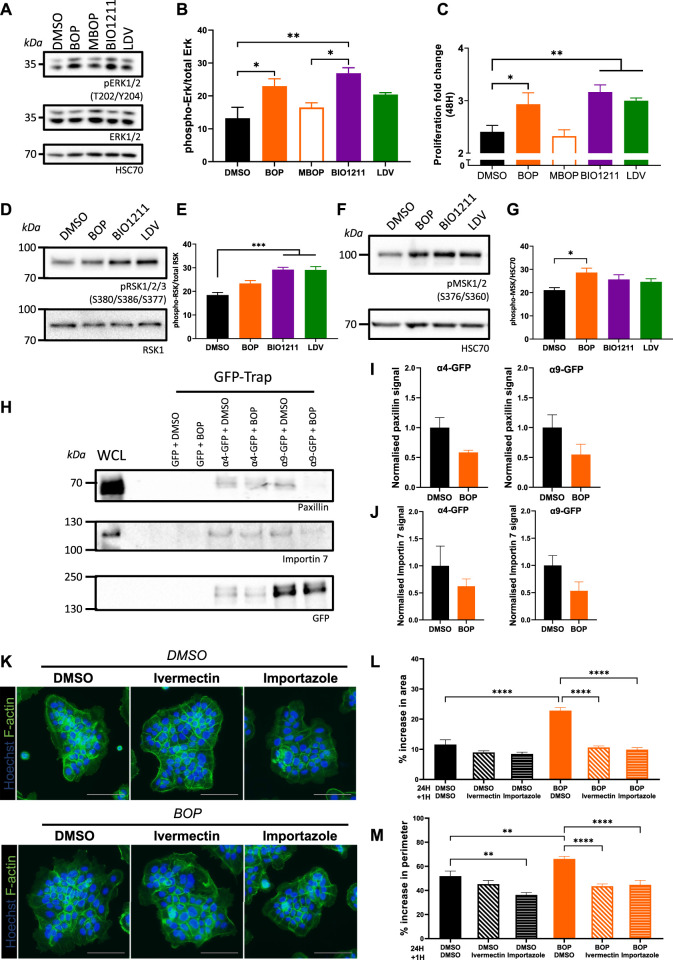
α4/α9 integrins negatively regulate ERK1/2 signaling. **(A)** Serum-starved keratinocytes were treated with DMSO (vehicle control), BOP, BIO1211, or LDV for 0.5 or 1 h before lysates were collected for Western blot analysis. Western blot probed for T202/Y204 phosphorylated ERK1/2, ERK1/2, and HSC70. **(B)** Western blot analysis representative of three independent experiments. Quantitative analysis of densitometric analysis of blots from three independent experiments showing mean ± s.e.m.; *p < 0.05, **p < 0.01. All other comparisons were not significant. **(C)** Quantitative analysis of proliferation fold change after 48 h of treatment was calculated using nuclei count. Data pooled from 12 wells per condition from four independent experiments showing mean ± s.e.m.; *p < 0.05, **p < 0.01. **(D)** Keratinocytes monolayers were treated with DMSO (vehicle control), BOP, BIO1211, or LDV for 0.5 h before lysates were collected for Western blot analysis. Western blot probed for S380/S386/S377 phosphorylated RSK1/2/3 and HSC70. **(E)** Quantitative analysis of densitometric analysis of blots from three independent experiments showing mean ± s.e.m.; ***p < 0.001. All other comparisons were not significant. **(F)** Keratinocytes monolayers were treated with DMSO (vehicle control), BOP, BIO1211, or LDV for 0.5 h before lysates were collected for Western blot analysis. Western blot probed for S376/S360 phosphorylated MSK1/2 and HSC70. **(G)** Quantitative analysis of densitometric analysis of blots from five independent experiments showing mean ± s.e.m.; *p < 0.05. All other comparisons were not significant. **(H)** Confluent monolayers of keratinocytes transiently expressing GFP control (GFP) or GFP tagged α4 integrin (α4-GFP) or GFP tagged α9 integrin (α9-GFP) for 48 h were treated with DMSO or BOP for 1 hour before whole cell lysates (WCL) were collected for immunoprecipitation with GFP-TRAP beads. Representative Western blot analysis of immunoprecipitation of α4-GFP and α4-GFP associated with paxillin, Importin 7, and GFP. **(I)** Quantification of fraction of paxillin bound to α4-GFP or α9-GFP following treatment with BOP, normalized to DMSO control. Data are pooled from three independent experiments showing mean ± s.e.m. Comparisons are not significant. **(J)** Quantification of fraction of Importin 7 bound to α4-GFP or α9-GFP following treatment with BOP, normalized to DMSO control. Data are pooled from three independent experiments showing mean ± s.e.m. Comparisons are not significant. **(K)** Widefield images of Ca^2+^ treated keratinocyte colonies treated with DMSO or BOP for 24 h prior to being treated with vehicle control (DMSO), Ivermectin, or Importazole for 1 h. Cells were then fixed and stained for nuclei and F-actin. Scale bar = 100 μm. Images representative of three independent experiments. **(L)** Quantitative analysis of percentage increase in area of actin protrusions over cortical actin ring. Data pooled from 30 colonies per condition from three independent experiments showing mean ± s.e.m.; ****p < 0.0001. All other comparisons were not significant. **(M)** Quantitative analysis of percentage increase in perimeter of actin protrusions over cortical actin ring. Data pooled from 30 colonies per condition from three independent experiments showing mean ± s.e.m.; **p < 0.005, ****p < 0.0001. All other comparisons were not significant.

Despite the role of α4 and α9 integrins in regulating keratinocyte adhesion and protrusion, neither integrin subunit colocalized to FA (Supplementary Figures S1C,D). We therefore hypothesized that α4 and α9 integrins may regulate shared signaling pathways to elicit these effects. α4/α9 integrins have previously been implicated in restricting ERK1/2 activity in keratinocytes (Modica et al., 2017; Danussi et al., 2011), and ERK1/2 can modulate cell motility by directly phosphorylating and activating several components of the actin cytoskeleton machinery (Tanimura and Takeda 2017). In agreement with these data, we found that short term inhibition of α4 and α9 integrins increased ERK1/2 phosphorylation compared to MBOP and our vehicle control (DMSO) (Figures 3A,B). As ERK is known to contribute to growth, we also analyzed proliferation; consistent with enhanced ERK1/2 activity, α4/α9 integrin inhibition also led to increased cell proliferation (Figure 3C). α4/α9 integrin inhibition also increased phosphorylation of downstream ERK1/2 effectors p90 ribosomal S6 kinase (RSK) (Figures 3D,E) and Mitogen and Stress Activated kinase (MSK) (Figures 3F,G). Active RSK can regulate cell adhesion to the ECM (Samson et al., 2019; Gawecka et al., 2012), whilst nuclear MSK can induce cell growth and through modulation of Histone H3, CREB, and ATF1 (Reyskens and Arthur 2016). This effect was cumulative, with an even more prominent increase in RSK and MSK phosphorylation following 6 h of α4/α9 integrin inhibition (Supplementary Figure S3). These data suggest that α4/α9 integrins collectively suppress ERK1/2 and downstream RSK and MSK activation, and this action may regulate the balance of cell proliferation and protrusion to enable efficient CCM.

To explore the potential mechanisms by which α4/α9 integrins could control the activity of ERK1/2 at the leading edge, keratinocytes transiently expressing α4-GFP and α9-GFP were subjected to GFP-trap immunoprecipitation. The isolated complexes were then fully resolved on an SDS PAGE gel, silver stained, and unique bands of interest containing possible binding partners were excised for LC-MS/MS analysis. Validation of these hits revealed paxillin bound to both integrins and confirmed that adhesion complexes were being isolated (Figure 3H). Furthermore, paxillin complexes were partially reduced following integrin inhibition (Figures 3H,I) supporting our previous data (Supplementary Figure S1C). The Heat Shock Protein (HSP) and Importin protein families were enriched in our LC-MS/MS screen (data not shown), which supports previous proteomics studies of integrin complexes (Byron et al., 2012). Validation of Importins as potential binding partners revealed Importin 7 was present in α4 and α9 integrin complexes, and this association showed a trend towards reduced binding following integrin inhibition (Figures 3H,J). Further analysis of the function of the integrin/Importin complex was investigated using the keratinocyte colony protrusion assay; colonies were co-treated with α4/α9 integrin and Importin inhibitors (Ivermectin and Importazole). Importin inhibition of colonies was well tolerated (Figure 3K), and α4/α9 integrin inhibition induced protrusions were rescued following Importin inhibition (Figure 3K,L,M). These data suggest the protrusive phenotype observed following α4/α9 integrin inhibition may be modulated through Importin release from integrin complexes, although further work will be needed to validate how this interaction is regulated.

### MSK1 has a Role in Regulating Actin Cytoskeleton Dynamics

**FIGURE 4:**
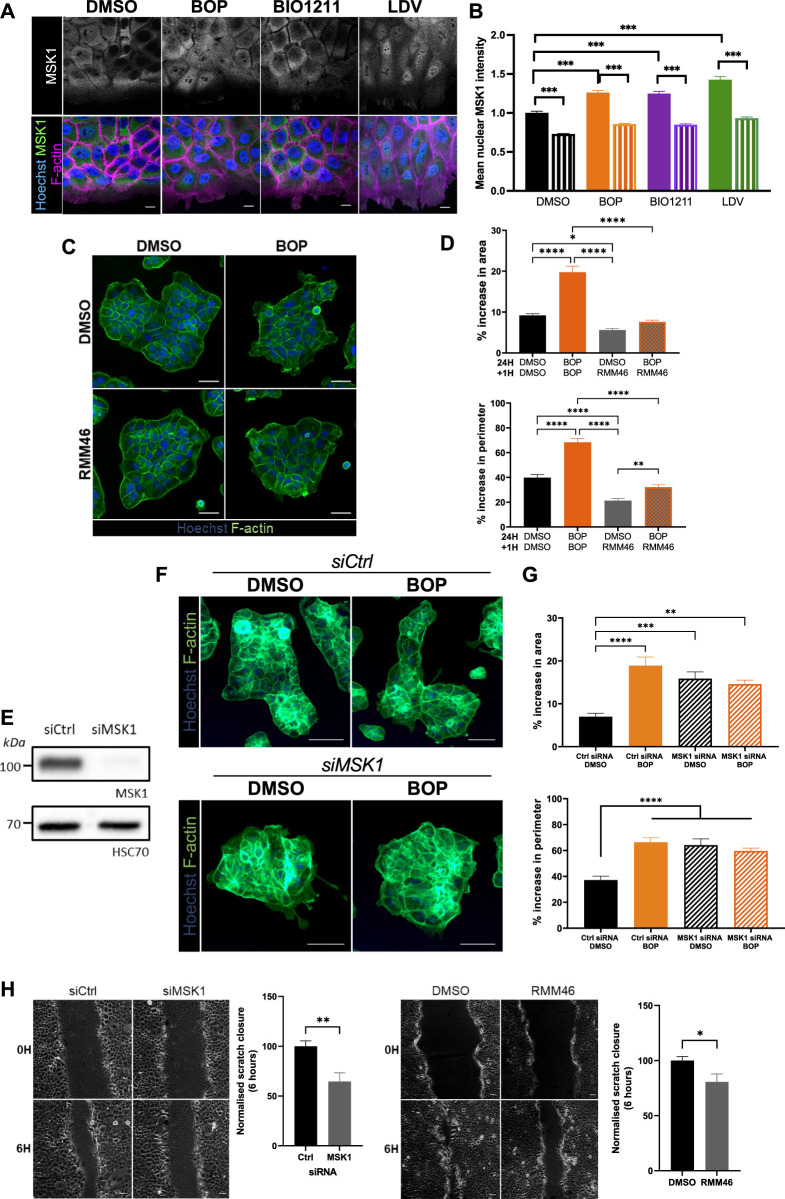
MSK1 regulation by α4/α9 integrins regulates protrusions and migration. **(A)** Maximum intensity projection of 20 μm Z stack of keratinocytes in scratch assay. Fixed cells were stained for nuclei, MSK1, and F-actin. Scale bar = 10 μm. Images representative of three independent experiments. **(B)** Quantitative analysis of mean nuclear MSK1 intensity. Solid bars show leader cells, hatched bars are MSK1 nuclear levels in follower cells. Data pooled from 20 fields of view per condition from three independent experiments showing mean ± s.e.m.; ****p < 0.0001. **(C)** Widefield images of Ca^2+^ treated keratinocyte colonies treated with vehicle control (DMSO) or BOP for 24 h prior to being cotreated with either DMSO or RMM46 for 1 h prior to fixing. Cells were then stained for nuclei and F-actin. Scale bar = 50 μm. Images representative of three independent experiments. **(D)** Quantitative analysis of percentage increase in area and perimeter of actin protrusions over cortical actin ring. Data pooled from 30 colonies per condition from three independent experiments showing mean ± s.e.m.; *p < 0.05, **p < 0.01, ****p < 0.0001. **(E)** Keratinocytes were transiently transfected with control siRNA pool (siCtrl) or MSK1 targeting siRNA pool (siMSK1) 48 h before lysates were collected for Western blot analysis. Western blot analysis from one independent experiment, probed for MSK1 and HSC70. **(F)** Widefield images of Ca^2+^ treated keratinocyte colonies treated with siCtrl or siMSK1 for 24 h prior to being treated with vehicle control (DMSO) or BOP for 24 h. Cells were then fixed and stained for nuclei and F-actin. Scale bar = 100 μm. Images representative of four independent experiments. **(G)** Quantitative analysis of percentage increase in area and perimeter of actin protrusions over cortical actin ring. Data pooled from 40 colonies per condition from four independent experiments showing mean ± s.e.m.; **p < 0.01, ***p < 0.001, ****p < 0.0001. All other comparisons were not significant. **(H)** Phase contrast images of keratinocyte scratch closure at 0 and 6 h. Keratinocytes were transfected with either control siRNA (siCtrl) or siRNA against MSK1 (siMSK1). 48 h post transfection, confluent keratinocyte monolayers were treated with 2 mM Ca^2+^, before being scratched with a pipette and imaged overnight. Confluent Ca^2+^ treated keratinocyte monolayers were scratched with a pipette tip 1 h prior to treatment with vehicle control (DMSO) or RMM46 and imaging overnight. Scale bar = 50 μm. Images representative of three independent experiments. Quantitative analysis of scratch closure after treatment for 6 h. Data pooled from nine movies per condition from three independent experiments showing mean ± s.e.m.; *p < 0.05, **p < 0.005.

MSK1 is a ubiquitously expressed serine/threonine protein kinase that localizes to the nucleus (Deak et al., 1998). However, recent evidence has demonstrated that MSK1 can be redistributed to the cytoplasm in response to specific stimuli (Beck et al., 2008), suggesting that MSK1 may have a currently undescribed role in the cytoplasm. As such, MSK1 activity has not yet been ascribed to the regulation of cellular migration. Surprisingly, we found that endogenously expressed MSK1 was predominantly localized to the cytoplasm in keratinocyte sheets (Figure 4A). Conversely, α4/α9 integrin inhibition increased MSK1 localization to the protrusive tips that extended beyond the leading edge (Figure 4A), and significantly increased MSK1 nuclear localization in leader cells, with significantly lower nuclear MSK1 levels in follower cells (Figures 4A,B). To determine whether this redistribution of MSK1 contributed to the α4/α9 integrin-dependent F-actin protrusion phenotype, we treated keratinocyte colonies with α4/α9 inhibitors in the presence or absence of an MSK1 inhibitor (RMM46). Imaging and analysis revealed that MSK1 inhibition suppressed the enhanced protrusions following α4/α9 integrin inhibition (Figures 4C,D). However, knockdown of MSK1 (Figure 4E) also enhanced the protrusive phenotype (Figures 4F,G), and hyperactivation of residual MSK1 following treatment with BOP did not induce a phenotypic rescue (Figures 4F,G). Furthermore, both MSK1 inhibition and knockdown had a detrimental effect on CCM (Figure 4H), suggesting that a more complex role for this kinase exists in the control of cell motility. Nevertheless, these findings demonstrate that MSK1 activity modulates cytoskeletal reorganization, and confirms that α4/α9 integrin suppression of MSK1 activity enables effective migration through the regulation of leading-edge protrusion dynamics.

## Discussion

**FIGURE 5:**
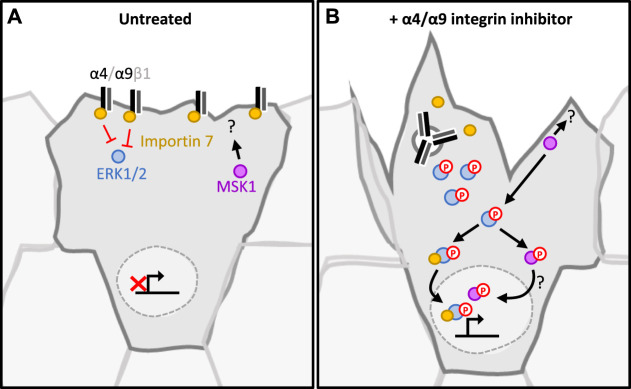
Proposed signaling pathway regulated by α4/α9 integrins in keratinocytes. **(A)** In untreated keratinocytes, α4 and α9 integrins complex with Importin 7. This suppresses ERK1/2 activation and signalling. As MSK1 and ERK1/2 phosphorylation is low, they are retained in the cytoplasm. This maintains the appropriate level of adhesion and protrusions required for collective cell migration. Proliferation rate is appropriately regulated, enabling lost cells to be replenished without impeding migrational persistence. **(B)** Integrins are internalised following inhibition, and this leads to the disruption of the integrin/Importin complex. This disruption induces ERK1/2 phosphorylation by a currently unknown mechanism. Active ERK1/2 phosphorylates and activates MSK1. This signalling cascade culminates with both MSK1 and ERK1/2 localising to the nucleus, which induces proliferation. MSK1 also accumulates in the protrusive tips, interacting with currently undefined cytoplasmic partners to induce cytoskeletal reorganisation at the leading edge.

Our study places α4β1 and α9β1 integrins as key regulators of keratinocyte CCM. Efficient wound healing requires a migratory front proximal to the wound to reinstate the barrier, whilst a distal ring of proliferating cells replenishes cells lost following injury (Park et al., 2017). Our data show that inhibition of α4/α9 integrins leads to increased ERK1/2 activation and downstream accumulation of nuclear MSK1. This finding suggests that α4 and α9 integrins may contribute to proliferation zonality during CCM through their localization and signaling; during wounding α4/α9 integrins relocalize to the leading-edge following engagement with components of the provisional ECM (for example, Tenascin-C). This altered distribution activates α4/α9 integrin mediated suppression of ERK1/2 signaling. ERK1/2 suppression could facilitate efficient movement at the migratory front by mediating transient attachment to ECM and cohesively organizing the cytoskeleton (summarized in Figure 5). Conversely, in follower cells, α4 integrins (sequestered at the cell junctions) and α9 integrins (sequestered within the cytoplasm) cannot suppress ERK1/2/MSK signaling, thus promoting normal proliferation rates. Furthermore, previous studies have indicated that α9 integrin regulates proliferation in skin (Singh et al., 2009), gut (Desloges et al., 1998), and lymphatic endothelial cells (Danussi et al., 2013). It will be interesting in future to determine if the pathways we have identified in keratinocytes extend to these other cell types.

Our data further demonstrate that inhibition of α4/α9 integrins increases ERK1/2 activation. This observation agrees with previous studies showing α4/α9 integrin binding to the ligand EMILIN-1 suppresses ERK1/2 signaling (Modica et al., 2017; Danussi et al., 2013; Danussi et al., 2011). Cytoplasmic ERK1/2 can play a role in cell motility by directly phosphorylating and activating several components of the actin cytoskeleton machinery (Tanimura and Takeda 2017), including the WAVE complex (Mendoza et al., 2011) and cortactin (Martinez-Quiles et al., 2004). Furthermore, downstream cytoplasmic ERK1/2 effector RSK can promote motility by activating myosin phosphatase to reduce actin contraction at the leading edge (Samson et al., 2019), and phosphorylating filamin A to inactivate β1 integrins (Gawecka et al., 2012). Furthermore, sustained ERK and MSK signaling induces proliferation (Lavoie, Gagnon, and Therrien 2020). As α4/α9 integrin inhibition results in activation of ERK1/2 signaling, it is likely that dysregulation of ERK1/2, RSK, and MSK may also impact on these pathways resulting in the phenotypes identified in our study.

Next, we sought to address how α4/α9 integrins regulate ERK1/2 signaling. Phosphorylated cytoplasmic ERK must associate with Importin 7 to cross the nuclear envelope (Chuderland, et al 2008). A body of evidence has previously implicated integrins in mediating ERK1/2 nuclear translocation (Hirsch et al., 2002; James et al., 2007) and cell cycle progression (Jeanes et al., 2012). This process offers the possibility that α4/α9 integrins could prevent ERK1/2 translocation by sequestering Importin 7 at the cell membrane. Alternatively, α4/α9 integrins may be regulating ERK activity through trans-dominant action over other integrins. Transdominance is the process by which integrins compete for ECM or cytoplasmic adaptor binding. Integrins with the greatest affinity for either suppress the level of signaling initiated by other integrins that also bind to those adaptors (Gonzalez et al., 2010). α9β1 has previously been shown to trans-dominantly repress α3β1 signaling during wound healing *in vitro* (DiPersio et al., 2016). Similarly, α4 integrin is trans-dominant over α5 integrins in melanoma cells (Moyano et al., 2003) and in oral squamous carcinoma cells (Zhang et al., 2004). α3β1 and α5β1 are expressed in keratinocytes (Watt and Jones 1993) and are known to induce ERK1/2 phosphorylation (Roovers et al., 1999; Manohar et al., 2004). Hence, α4/α9 integrin inhibition may increase ERK1/2 activation by initiating signaling pathways downstream of these other β1 integrin containing heterodimers. We hypothesize that α4/α9 integrins may therefore locally regulate ERK1/2 activity and nuclear import to prevent proliferation and instead promote migration at the leading edge.

As α4/α9 integrin inhibition suppressed CCM in our study, this inferred that MSK (initially identified in our kinase screen) may have a previously undescribed role in migration. Interestingly, MSK1 inhibition suppressed residual protrusions at the boundary of keratinocyte colonies. Spatiotemporal co-ordination of protrusive activity is essential for migration (Pankov et al., 2005; Dang et al., 2013), and our data suggest that a basal level of MSK signaling is required for optimal cytoskeleton dynamics to permit efficient migration. In support of this interpretation, MSK inhibition and deletion both suppressed CCM. Unexpectedly, MSK1 knockdown also increased protrusions, which suggests that MSK1 may scaffold other protein(s) at the leading edge, which in turn restricts membrane protrusions. This possibility could be supported by the observation that MSK1 was enriched in the protrusive tips of keratinocytes following α4/α9 integrin inhibition. Active MSK1 has principally been studied in the nucleus, and potential cytoplasmic interaction partners or substrates are currently unknown. This topic would be an interesting area for future study and may reveal additional MSK1 dependent targets that play a role in actin dynamics and motility.

In summary, we have identified α4 and α9 integrins as key regulators of CCM in keratinocytes. This study also identifies a previously undescribed role for MSK in the regulation of cytoskeletal dynamics during keratinocyte migration. These findings contribute towards our understanding of the regulation of keratinocyte migration within wound healing, and therefore offer interesting insights into the signaling cascades regulated by this integrin subfamily.

## Data Availability

The original contributions presented in the study are publicly available. This data can be found here: ProteomeXchange accession number: PXD027944.

## Supplementary Material

Supplementary Figure S1
**(A, B)** Cross-section of human skin. Ep, Epidermis. De, Dermis. Dermal-epidermal boundary highlighted with orange dashed line. **(A)** Section stained for α4 integrin and nuclei. Scale bar = 100 µm. **(B)** Section stained for α9 integrin and nuclei. Scale bar = 100 µm. **(C)** Single confocal Z image at the basal plane at leading edge. Confluent Ca^2+^ treated keratinocyte monolayers were scratched with a pipette tip 1 h prior to treatment with vehicle control (DMSO) or BOP for 1 h. Fixed cells were then stained for nuclei, paxillin, integrin, and F-actin. Scale bar = 10 μm. Images representative of two independent experiments. **(D)** Confluent Ca^2+^ treated keratinocyte monolayers were scratched with a pipette tip 1 h prior to fixing. Stained for nuclei, focal adhesion marker (talin or vinculin), α9 integrin, and F-actin. Scale bar = 10 μm. White box indicates magnified ROI. Images representative of three independent experiments. **(E)** Binding of BOP-JF549 to α4β1 and α9β1. Dissociation constants are reported. Affinity for both α4β1 and α9β1 is in the low nanomolar range, and marginally higher for α9β1. Protein concentration is shown using a logarithmic scale. Data shown represent three independent experiments and are expressed as mean fluorescence polarization ±s.e.m.

Supplementary Figure S2
Blocking α4/α9 integrins activity induces α4/α9 integrin internalization but does not degrade target integrins. (A) Single confocal Z image in the central plane of confluent Ca^2+^ treated keratinocyte monolayer transiently transfected with GFP tagged α4 integrin 48 h prior to imaging. Cells were imaged using time-lapse confocal microscopy, and after 10 min cells were treated with BOP-JF646 for 1.5 h. Time displayed in white text as hours:minutes. Scale bar = 10 μm. Images representative of two independent experiments. **(B)** Quantitative analysis of internalized α4 integrin in follower cells. Confluent Ca^2+^ treated keratinocyte monolayers were scratched with a pipette tip 1 h prior to treatment with vehicle control (DMSO), BOP, BIO1211, or LDV for 1 h. Fixed cells were then stained for α4 integrin. The percentage of α4 integrin signal in the cytoplasm compared to the whole cell was calculated. Data pooled from 30 cells per condition from three independent experiments showing mean ± s.e.m.; **p < 0.01. All other comparisons were not significant. **(C)** Single confocal Z image at the basal plane of keratinocytes in scratch assay. Confluent Ca^2+^ treated keratinocyte monolayers were scratched with a pipette tip 1 h prior to treatment with vehicle control (DMSO), BOP, BIO1211, or LDV for 1 h. Fixed cells were stained for nuclei, α9 integrin, and F-actin. Scale bar = 10 μm. Images representative of three independent experiments. **(D)** Quantitative analysis of α9 integrin signal in leader cells. Data pooled from 15 fields of view per condition from three independent experiments showing mean ± s.e.m.; ***p < 0.001. All other comparisons were not significant. **(E)** Keratinocytes were treated with either vehicle control (DMSO), BOP, BIO1211, or LDV for 24 h before lysates were collected for Western blot analysis. Western blot analysis representative of three independent experiment, probed for α4 integrin, α9 integrin, β1 integrin, and HSC70. No change in integrin levels was observed. **(F)** Keratinocytes were treated with either vehicle control (DMSO), cycloheximide, MG132, or leupeptin for up to 24 h before lysates were collected for Western blot analysis. Western blot analysis representative of two independent experiment, probed for p53, TNF-R1, α4 integrin, α9 integrin, α2 integrin, and HSC70. Quantitative analysis of α4 and α9 integrin blots. Densitometric analysis from two independent experiments showing mean ± s.e.m; no significance was calculated. **(G)** Keratinocytes were treated either vehicle control (DMSO), MG132, or leupeptin for up to 48 h before lysates were collected for Western blot analysis. Western blot analysis representative of two independent experiment, probed for α4 integrin, α9 integrin and GAPDH. Quantitative analysis of α4 and α9 integrin blots. Densitometric analysis from two independent experiments showing mean ± s.e.m; no significance was calculated.

Supplementary Figure S3 
Kinase array reveals novel targets regulated by α4β1/α9β1 integrins. Ca^2+^ treated keratinocyte colonies treated with DMSO (vehicle control), BOP, BIO1211, or LDV for 0.5 or 6 h. Lysates were collected for Human Phospho-Kinase Array Analysis. Phosphorylation of proteins in samples was calculated using densiometric analysis. Signal intensities were normalized to Heat Shock Protein 60 (HSP60) internal control, and the fold change in phosphorylation relative to DMSO was calculated.

## Abbreviations

BOP, N-(Benzenesulfonyl)-L-prolyl-L-O-(1-pyrrolidinylcarbonyl) tyrosine sodium salt; CCM, collective cell migration; ERK1/2, extracellular signal-regulated kinase 1/2; FA, focal adhesions. FP, Fluorescence Polarization assay; FOV, Fields of view; HSC70, Heat Shock Cognate 70; HSP, Heat Shock Protein; LC-MS/MS, Liquid chromatography with tandem mass spectrometry analysis; MSK1, Mitogen and Stress Activated Kinase 1; RSK, p90 ribosomal S6 kinase.
