# Supplementary material for: α4/α9 Integrins Coordinate Epithelial Cell Migration Through Local Suppression of MAP Kinase Signaling Pathways
Source: Front Cell Dev Biol. 2021 Nov 25;9:750771. doi: 10.3389/fcell.2021.750771 (PMC8655878; doi:10.3389/fcell.2021.750771)
Supplement: Supplementary file 1 [file Image2.pdf]

Supplementary Figure 2

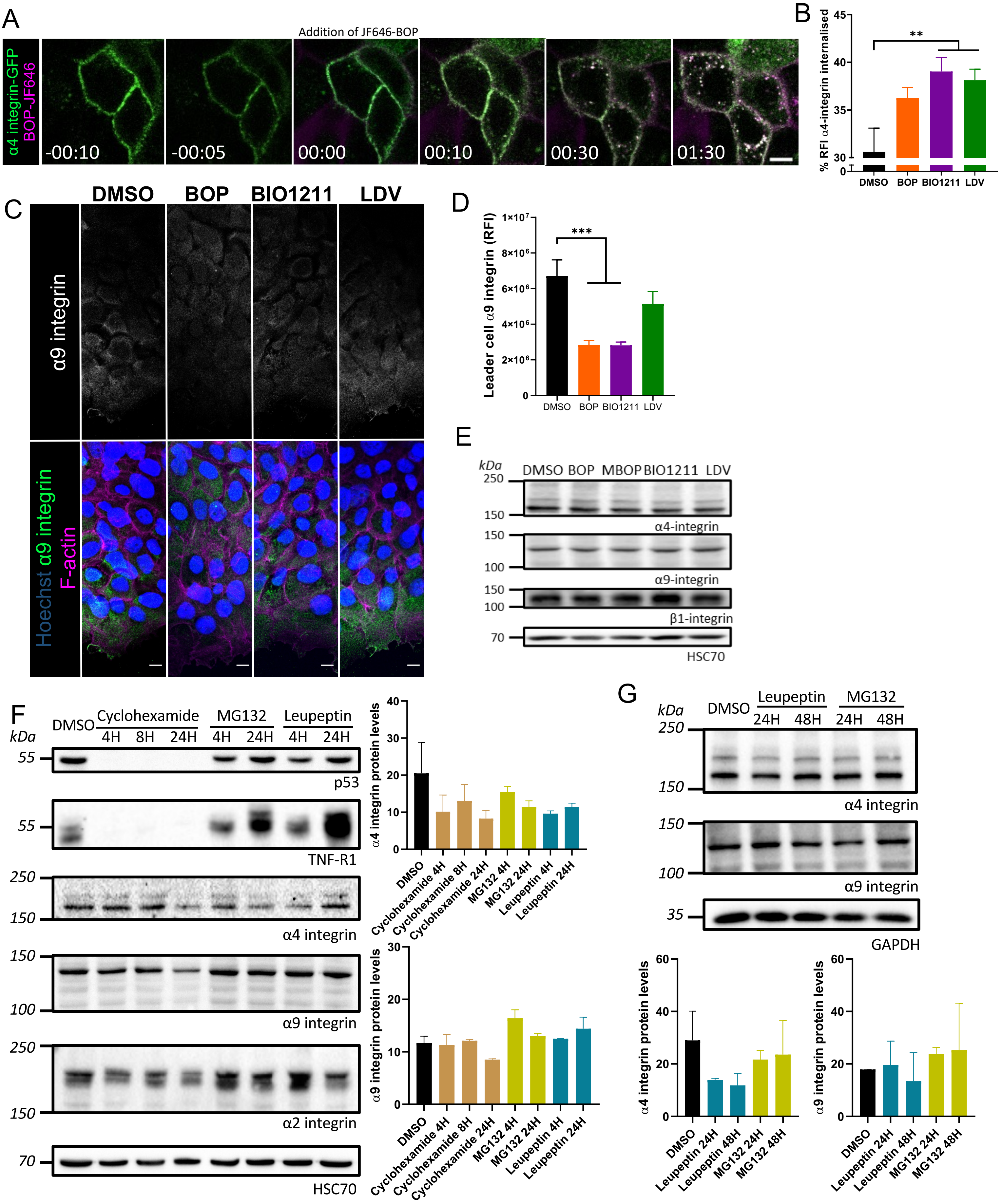

Supplementary Figure 2: Blocking α4/α9 integrins activity induces α4/α9 integrin internalization but does not degrade target integrins. (A) Single confocal Z image in the central plane of confluent Ca^{2+} treated keratinocyte monolayer transiently transfected with GFP tagged α4 integrin 48 hours prior to imaging. Cells were imaged using time-lapse confocal microscopy, and after 10 minutes cells were treated with BOP-JF646 for 1.5 hours. Time displayed in white text as hours:minutes. Scale bar = 10μm. Images representative of two independent experiments. **(B)** Quantitative analysis of internalized α4 integrin in follower cells. Confluent Ca^{2+} treated keratinocyte monolayers were scratched with a pipette tip 1 hour prior to treatment with vehicle control (DMSO), BOP, BIO1211, or LDV for 1 hour. Fixed cells were then stained for α4 integrin. The percentage of α4 integrin signal in the cytoplasm compared to the whole cell was calculated. Data pooled from 30 cells per condition from three independent experiments showing mean ± s.e.m.; ** $p < 0.01$. All other comparisons were not significant. **(C)** Single confocal Z image at the basal plane of keratinocytes in scratch assay. Confluent Ca^{2+} treated keratinocyte monolayers were scratched with a pipette tip 1 hour prior to treatment with vehicle control (DMSO), BOP, BIO1211, or LDV for 1 hour. Fixed cells were stained for nuclei, α9 integrin, and F-actin. Scale bar = 10μm. Images representative of three independent experiments. **(D)** Quantitative analysis of α9 integrin signal in leader cells. Data pooled from 15 fields of view per condition from three independent experiments showing mean ± s.e.m.; *** $p < 0.001$. All other comparisons were not significant. **(E)** Keratinocytes were treated with either vehicle control (DMSO), BOP, BIO1211, or LDV for 24 hours before lysates were collected for Western blot analysis. Western blot analysis representative of three independent experiment, probed for α4 integrin, α9 integrin, β1 integrin, and HSC70. No change in integrin levels was observed. **(F)** Keratinocytes were treated with either vehicle control (DMSO), cycloheximide, MG132, or leupeptin for up to 24 hours before lysates were collected for Western blot analysis. Western blot analysis representative of two independent experiment, probed for p53, TNF-R1, α4 integrin, α9 integrin, α2 integrin, and HSC70. Quantitative analysis of α4 and α9 integrin blots. Densitometric analysis from two independent experiments showing mean ± s.e.m; no significance was calculated. **(G)** Keratinocytes were treated either vehicle control (DMSO), MG132, or leupeptin for up to 48 hours before lysates were collected for Western blot analysis. Western blot analysis representative of two independent experiment, probed for α4 integrin, α9 integrin and GAPDH. Quantitative analysis of α4 and α9 integrin blots. Densitometric analysis from two independent experiments showing mean ± s.e.m; no significance was calculated.
