# Supplementary material for: α4/α9 Integrins Coordinate Epithelial Cell Migration Through Local Suppression of MAP Kinase Signaling Pathways
Source: Front Cell Dev Biol. 2021 Nov 25;9:750771. doi: 10.3389/fcell.2021.750771 (PMC8655878; doi:10.3389/fcell.2021.750771)
Supplement: Supplementary file 2 [file Image3.pdf]

Supplementary Figure 3

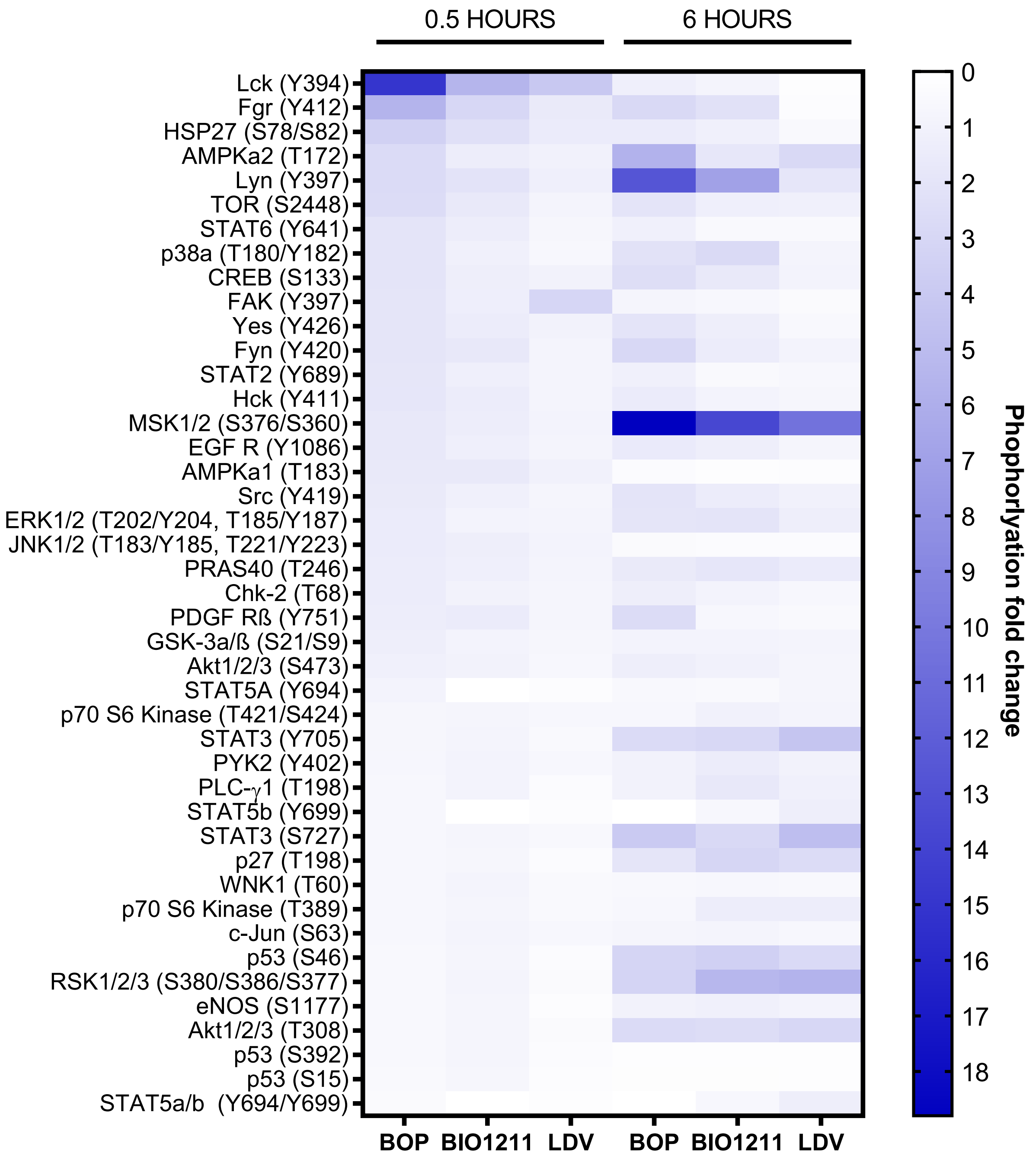

Supplementary Figure 3: Kinase array reveals novel targets regulated by α4β1/α9β1 integrins. Ca^{2+} treated keratinocyte colonies treated with DMSO (vehicle control), BOP, BIO1211, or LDV for 0.5 or 6 hours. Lysates were collected for Human Phospho-Kinase Array Analysis. Phosphorylation of proteins in samples was calculated using densiometric analysis. Signal intensities were normalized to Heat Shock Protein 60 (HSP60) internal control, and the fold change in phosphorylation relative to DMSO was calculated.
