# Supplementary material for: α4/α9 Integrins Coordinate Epithelial Cell Migration Through Local Suppression of MAP Kinase Signaling Pathways
Source: Front Cell Dev Biol. 2021 Nov 25;9:750771. doi: 10.3389/fcell.2021.750771 (PMC8655878; doi:10.3389/fcell.2021.750771)
Supplement: Supplementary file 3 [file Image1.pdf]

Supplementary Figure 1

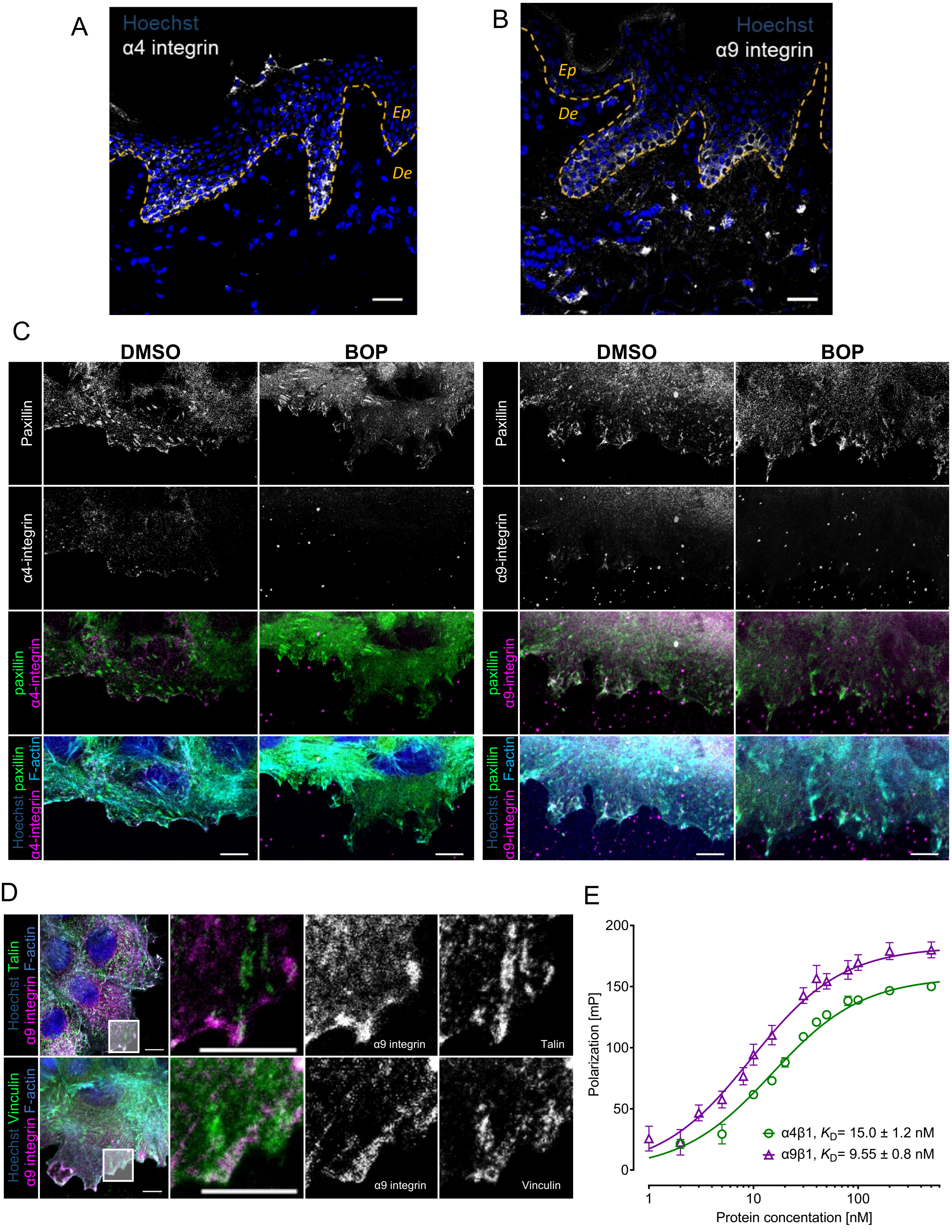

Supplementary Figure 1: (A, B) Cross-section of human skin. Ep, Epidermis. De, Dermis. Dermal-epidermal boundary highlighted with orange dashed line. **(A)** Section stained for α4 integrin and nuclei. Scale bar = 100µm. **(B)** Section stained for α9 integrin and nuclei. Scale bar = 100µm. **(C)** Single confocal Z image at the basal plane at leading edge. Confluent Ca^{2+} treated keratinocyte monolayers were scratched with a pipette tip 1 hour prior to treatment with vehicle control (DMSO) or BOP for 1 hour. Fixed cells were then stained for nuclei, paxillin, integrin, and F-actin. Scale bar = 10µm. Images representative of two independent experiments. **(D)** Confluent Ca^{2+} treated keratinocyte monolayers were scratched with a pipette tip 1 hour prior to fixing. Stained for nuclei, focal adhesion marker (talin or vinculin), α9 integrin, and F-actin. Scale bar = 10µm. White box indicates magnified ROI. Images representative of three independent experiments. **(E)** Binding of BOP-JF549 to α4β1 and α9β1. Dissociation constants are reported. Affinity for both α4β1 and α9β1 is in the low nanomolar range, and marginally higher for α9β1. Protein concentration is shown using a logarithmic scale. Data shown represent three independent experiments and are expressed as mean fluorescence polarization ± s.e.m.
